# Eyelid Cosmetic Enhancements and Their Associated Ocular Adverse Effects

**Published:** 2019

**Authors:** Maliha MASUD, Majid MOSHIRFAR, Tirth J. SHAH, Aaron T. GOMEZ, Michele R. AVILA, Yasmyne C. RONQUILLO

**Affiliations:** 1 University of Utah, Salt Lake City, Utah, USA; 2 John A. Moran Eye Center, Dept. of Ophthalmology and Visual Sciences, University of Utah School of Medicine, Salt Lake City, Utah, USA; 3 Utah Lions Eye Bank, Murray, Utah, USA; 4 Hoopes, Durrie, Rivera Research, Hoopes Vision, Draper, Utah, USA; 5 College of Medicine, University of Arizona, Phoenix, Arizona, USA; 6 University of Texas, Rio Grande Valley, Edinburg, Texas, USA

**Keywords:** Eyelash Extensions, Eyelid Tattooing, Eyelash dyeing, Reconstructive Surgical Procedures

## Abstract

Numerous cosmetic enhancements and augmentations to the natural appearance of the periorbital area are readily available today. Due to the increasing popularity of these cosmetic procedures, it is important for ophthalmologists to be aware of their potential risks, complications and adverse effects. The aim of this literature review was to introduce some of the most common ocular cosmetic enhancements and provide a comprehensive overview of their associated adverse effects reported in various medical journals. PubMed, Embase, and Google Scholar were used to identify articles related to the following ocular cosmetic procedures; eyelash extensions, permanent eyelid tattooing, and eyelash dyeing. The most common complication associated with eyelash extensions was allergic blepharitis (79%). Allergic granulomatous reactions were the predominant complication in patients who underwent eyelid tattooing (56%). Besides, 60% of subjects who underwent eyelash dyeing experienced allergic contact dermatitis as the most common adverse effect. Although millions of these procedures are performed annually without any adverse effects, reports of complications continue to increase in the literature. Knowledge of the possible adverse effects associated with these enhancements is important for eye care providers and licensed estheticians to be aware of given both the direct and indirect effects they may have on ocular health and visual outcomes.

## INTRODUCTION

Cosmetic enhancements, both permanent and semi-permanent, have shaped society for centuries. Ancient cultures such as the Incans, Aztecs and Egyptians paved the way for later civilizations regarding manipulation of different substances for ocular cosmetic use [1]. New techniques have contributed to the evolution of these enhancements over time. Some of the most popular ocular enhancements today include eyelash extensions, permanent eyelid tattooing and eyelash dyeing. Eyelash extensions involve adhesion of artificial lash fibers to the base of the natural lash via potent acrylate glues [2]. This cosmetic alteration is performed worldwide and with similar acrylate adhesives, found to contain toxic formaldehyde-emitting compounds. Following exposure to substances used for adhesion and mechanical limitations of the false lashes, adverse effects are reported including a variety of allergic reactions and damages to the ocular surface [2].

Eyelid tattooing, or blepharopigmentation, is another widely-practiced cosmetic procedure. Using a round-tip needle and various inks, pigment is deposited into the superficial dermis and along the cilia of the eyelid to enhance the shape of the eye [3, 4]. Due to the fragile nature of this procedure and unknown chemical toxicity of the tattoo inks, mild to severe adverse reactions were reported following application [3, 5].

Eyelash dyeing employs semi-permanent darkening of the lash line and lashes using a combination of hair dye and a hydrogen peroxide developer [6]. The patient’s eyes are first prepped with petroleum jelly to the periorbital surface, to protect from excess dye, after which the topical dye mixture is applied to the lashes and removed soon after [6]. The dyes most commonly used during eyelash dyeing contain P-phenylene diamine and black henna which have been reported to cause a number of allergy-related ocular damage and other more severe ocular diseases [7, 8].

Given the growing popularity of these cosmetic enhancements, reported complications, and consequences related to these procedures are increasing. Hence, it is important for all eye care providers to be familiar with possible complications associated with these techniques. 

## METHODS

The resources utilized included the electronic database Medline (PubMed; https://pmid:), Embase, and Google Scholar. Keywords used in the search included eyelash, eyelash extension, lash extension, cyanoacrylate, cyanoacrylate injury, cyanoacrylate eye, blepharopigmentation, eyelid tattoo, eye tattoo, permanent eyeliner, permanent cosmetics, eye tattoo injury, tattoo dermatitis, ocular dermatitis, eyelash tint, eyelash dye, black henna eye, P-phenylene diamine, PPD eye, hair dyes, hydrogen peroxide eye, mascara dye, globe penetration and eyeball tattoo injury.

All English-language literature review articles and case reports published from May 1934 through December 2017 were reviewed in this study. There were collectively 77 articles on these topics. By filtering them, 54 were found to be case reports and adverse events. Of those 54 articles, 19 described adverse events of eyelash dyeing, 17 regarding eyelid tattooing and 18 about eyelash extension. Studies were excluded if the method of injury was not related to eyelash extensions, eyelid tattooing or eyelash dyeing. No other exclusion criteria were applied.

Eyelash Extensions

By definition, eyelash extensions are the adhesion of false lashes to the base of natural lashes. They are heavily used in countries around the world including the United States, Nigeria, Ghana, Japan and China [2]. Abah et al [2] studied females aged 10 to 39 who actively used eyelash extensions and found the main reason they chose to alter their lashes was to enhance natural beauty and increase femininity. However, there are several adverse effects associated with this procedure. For instance, respondents in another study reported adverse effects following eyelash extensions, including dry eyes, burning sensations, lid swelling, pain, and redness following application of the eyelash extensions [9]. Recent studies have reported that 73.3% of patients experienced ocular side effects after the application of eyelash extensions including itching (45.8%), redness (45.5%), pain (43.9%) and heavy eyelids (41.6%) [2]. Failure to treat those reactions most often leads to other serious ocular disorders including contact dermatitis, toxic conjunctivitis, conjunctival erosion and allergic blepharitis [10]. Many studies have investigated the cause of these ocular responses and narrowed down the sources to the lash glue adhesive and to mechanical limitations of the lash extensions themselves. The glue, which provides adhesion for the false lash to the natural lash, is primarily cyanoacrylate-based and contains latex and ammonia. The glue has shown to be a high formaldehyde-emitting product and is common in almost every country [2]. These products with allergy provoking substances have been implicated multiple times in producing mild to severe contact dermatitis, keratoconjunctivitis and blepharitis following contact with the acrylate [10, 11].

There are several mechanical consequences to eyelash extensions as well. These include lagophthalmos during sleep which increases corneal exposure and dryness, collection of bacteria under the lash bed causing microbial infection, constraints to physical hygiene and cleansing of the lids leading to infection, and calcification of the lash base causing scratching of the corneal surface [9]. Gel pads are often applied under the lower lash line during the application of eyelash extensions. These pads hold down the lower lashes and protect the sensitive skin under the eyes [12]. The pads have shown to contain the preservative methylisothiazolinone (MI) which can also irritate the periorbital area and cause mild to severe allergic reactions [12].

**Figure 1:**
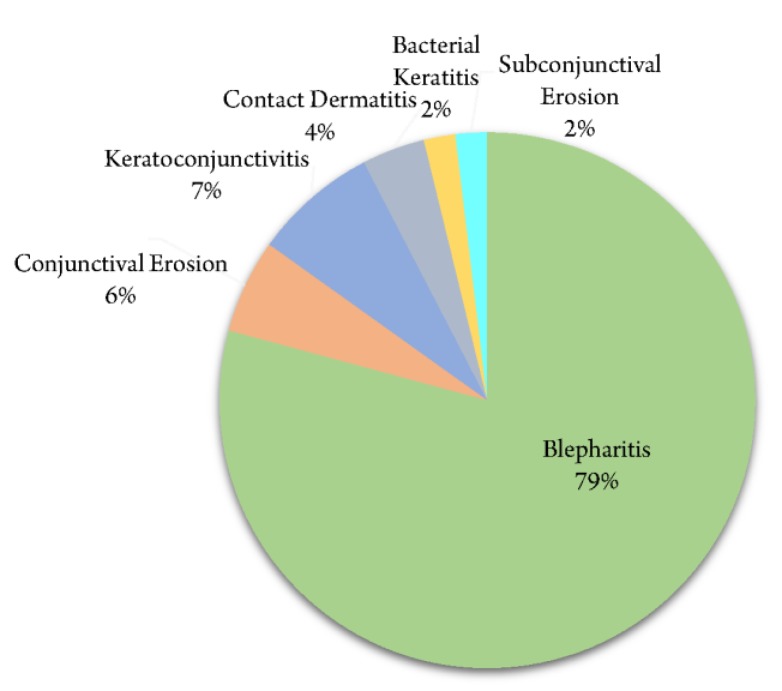
Calculated Percentages for All rReported Complications of Eyelash Extensions Published in Medical Journals. Methodology for Findings Include Quantifying the Number of Adverse Events Reported in the Literature per Complication and Calculating the Percentage of Prevalence Given the Total.

In the literature review, there were 42 cases of allergic blepharitis, 4 keratoconjunctivitis, 3 conjunctival erosion, 2 contact dermatitis, 1 bacterial keratitis, and 1 subconjunctival erosion associated with eyelash extensions (Fig. 1) [10, 13, 14]. While there is a paucity of literature on the adverse effects of eyelash extensions, the number of case reports detailing complications are on the rise. 

Permanent Eyelid Tattooing

Blepharopigmentation (eyelid tattooing) is another cosmetic enhancement gaining popularity in the modern world. It is most often performed to give the appearance of permanent eyeliner on the upper and/or lower lids. The procedure consists of distribution of pigments along the eyelid cilia and into the superficial dermis to enhance the appearance of both the upper and lower lash lines [3]. The procedure is performed using a round-tip needle and various ink pigments frequently containing copper, aluminum, and titanium [4].

**Figure 2:**
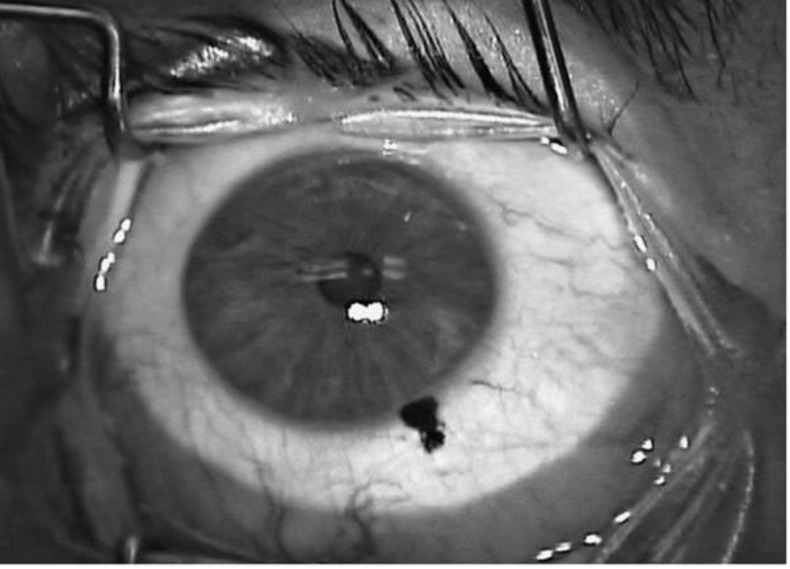
The Right Eye: Inadvertent Pigmentation of the Limbus following Misapplication and Ocular Penetration during Permanent Eyelid Tattooing. Courtesy of Dr. Majid Moshirfar.

There can be many complications associated with this procedure even when it is performed by a licensed tattoo artist or esthetician. The most common adverse reactions to eyelid tattooing include dermatitis, allergic blepharitis, and tear film instability [3]. These responses stem from the body reaction to the foreign substance, allergic reactions to the pigments, and misapplication of the ink [4]. Improper application of the pigment (Fig. 2), poor distribution of the pigment and pigment fanning in the tarsus can collectively and severely damage the ocular surface and periorbital area [3]. Severe complications following application mistakes can include corneal erosion, corneal staining, and meibomian gland loss. These complications may result in long-term issues for the patient and possible decline of visual acuity. 

Chemical toxicity of the ink has also been reported to cause ocular surface abnormalities which may be exacerbated through meibomian gland dysfunction and recurrent inflammation [3, 5]. These ocular complications arise from lysis of cells with phagocytosed pigment following tattoo pigment-induced mast cell activation [4]. Eyelid tattooing most often requires multiple applications to fulfill the desired outcome, which heightens the risk of the complications due to recurrent allergic reactions and damage to the ocular surface [3].

There is concern that these tattoo inks are not FDA approved and specific brands’ pigments have been deemed harmful in an FDA-issued consumer alert [4]. Recent findings have also reported delayed hypersensitivity inflammatory reactions and ocular vessel damage due to tattoos applied to arms and other distant body parts [4]. Studies have also shown long-term tolerance to the impure ink pigments and suggest that the severity of reactions may also depend on needle penetration depth and patient allergy reaction [15].

**Figure 3:**
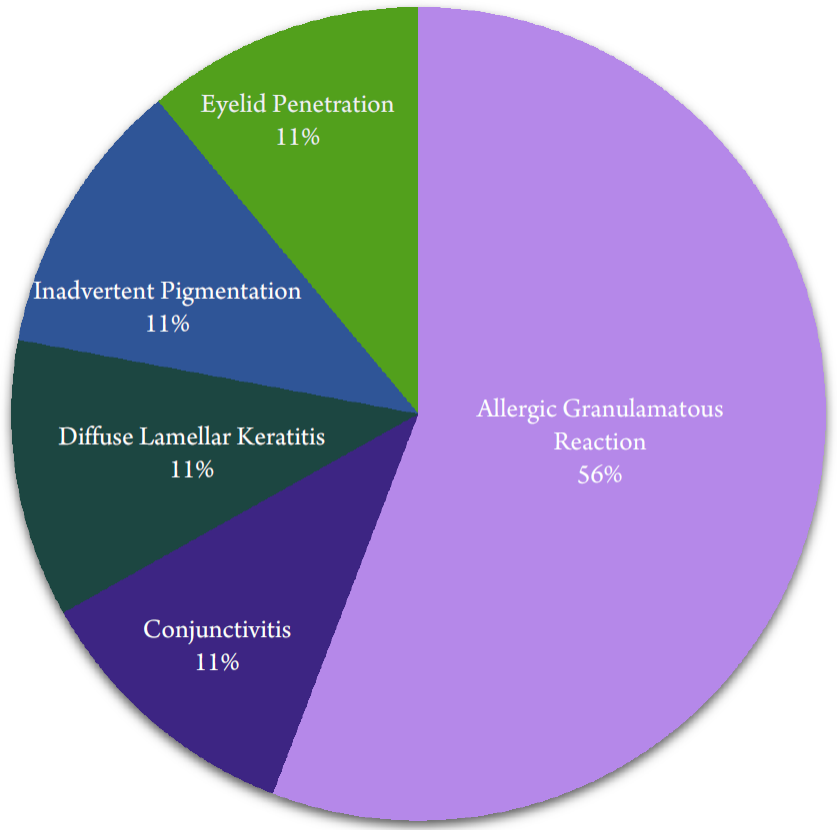
Calculated Percentages for all Reported Complications of Permanent Eyelid Tattooing Published in Medical Journals.

In the literature, there were 5 cases of allergic granulomatous reactions, 1 conjunctivitis, 1 late-onset diffuse lamellar keratitis, 1 inadvertent pigmentation of the limbus, and 1 eyelid penetration related to eyelid tattooing (Fig. 3) [3, 4, 16, 17].

Eyelash Dyeing

Another form of eyelash enhancement that is gaining popularity is eyelash dyeing. Eyelash dyeing mimics the lash-darkening principles of mascara but with semi-permanent effects [6]. 

The procedure consists of cleansing the area, applying petroleum jelly on the surrounding surfaces for protection, mixing the dye with a developer containing hydrogen peroxide, applying the mixture to the lashes, and removing the excess dye [6]. It should be performed by a qualified professional.

Although normally performed by licensed estheticians, eyelash tinting products are available to the general public. This allows for increased risk of improper use and complications. A case study performed in 1998-2002 in an out-patient eye clinic documented the number of patients with allergy-related ocular diseases and their associated causes. In this five-year study (n:272), 63.2% of the patients had used eyelash dyes and approximately 80% reported adverse conditions following application [18].

**Figure 4:**
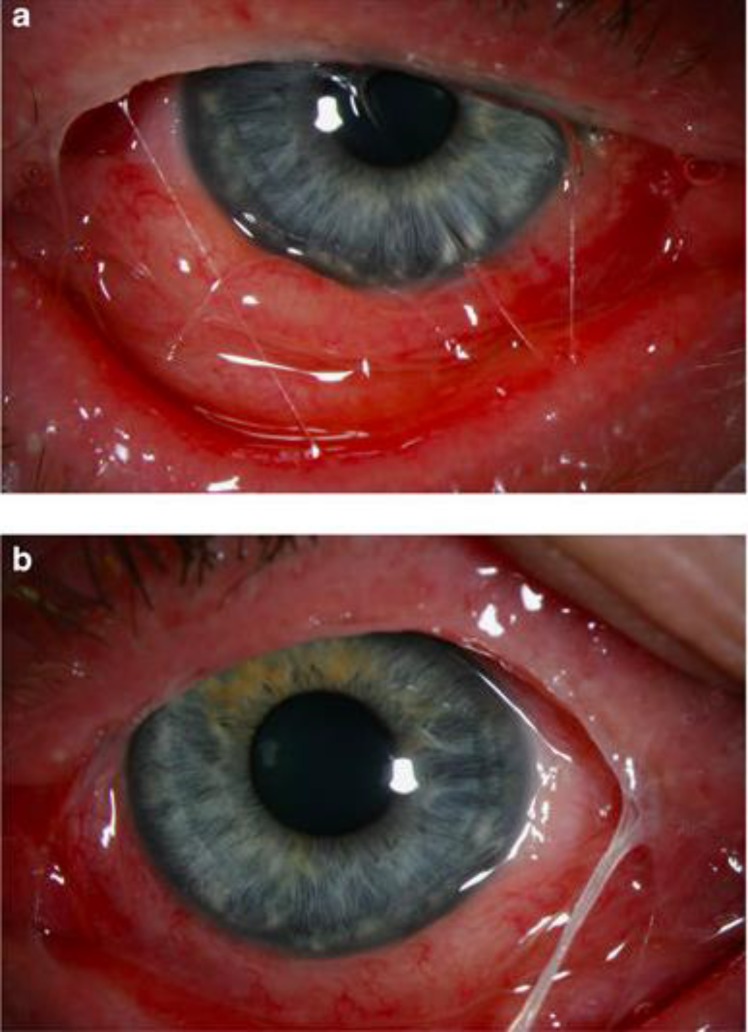
The Right Eye (a) and the Left Eye (b) Showing Lid Swelling, Conjunctival Chemosis and Congestion With Ropy Discharge Following Eyelash Dyeing. Courtesy of M.A Awan

The dyes used during the lash-tinting treatment most commonly contain stearic acid, acetyl alcohol, triethanolamine and 2-chloro-p-phenylemediamine [7]. These dyes have been reported to cause severe blepharoconjunctivitis, contact dermatitis, edema of the lids, and periorbital dermatitis [19, 20]. P-phenylene diamine (PPD) is the most common sensitizer in hair dyes and is extremely irritating to the skin [21]. Few case reports have discussed the widespread effects of PPD following eyelash dyeing. These adverse effects include loss of eyelashes and inflammatory responses spreading to the lids, conjunctiva and surrounding skin (Fig. 4) [21, 22]. However, some of the most severe reactions involving the ocular surface included erosions and ulcerations of the cornea [19].

Eyelash dyes have also been incorporated into many cosmetic products. In a case report from 2006, a professional eyelash tint product, Revlon Professional Roux Lash and Brow Tint (Colomer USA Corp, New York, New York, the USA), was studied in three patients for the effects on the ocular surface and periorbital area [23]. This specific product, unlike others on the market, is designed for continued application to the lashes. Following self-application and long-term use of the eyelash tint, deposition of silver pigment, the major component of the product, was found on the lid margin, conjunctiva and in one case, in the basement membrane and superficial substantia propria of the conjunctiva [23]. The study also revealed how continued usage of the product and others like it may result in corneal and conjunctival argyrosis due to prolonged exposure to silver [23]. Black henna is another product commonly used for hair dye. It is made by mixing the natural powdered leaves of the mignonette tree with PPD and a hydrogen peroxide base. Black henna has been reported to result in periocular/periorbital contact dermatitis and in one case blepharoconjunctivtis [8]. This product provides adequate eyelash dyeing outcomes and is easily accessible to the public. Even though it is derived from a natural source, it can be equally as harmful as its professional eyelash dye counterparts. 

**Figure 5:**
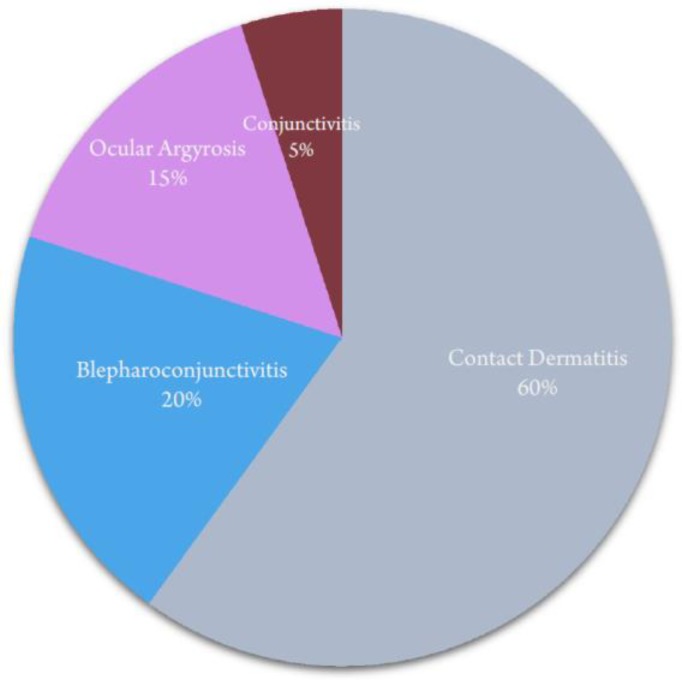
Calculated Percentages for All Reported Complications of Eyelash Dyeing Published in Medical Journals.

In the literature, there were 12 cases of allergic contact dermatitis, 4 blepharoconjunctivitis, 3 ocular argyrosis, and 1 conjunctivitis all associated with the use of eyelash dyes (Fig. 5) [6-8, 20, 22, 24-26].

## DISCUSSION

**Figure 6:**
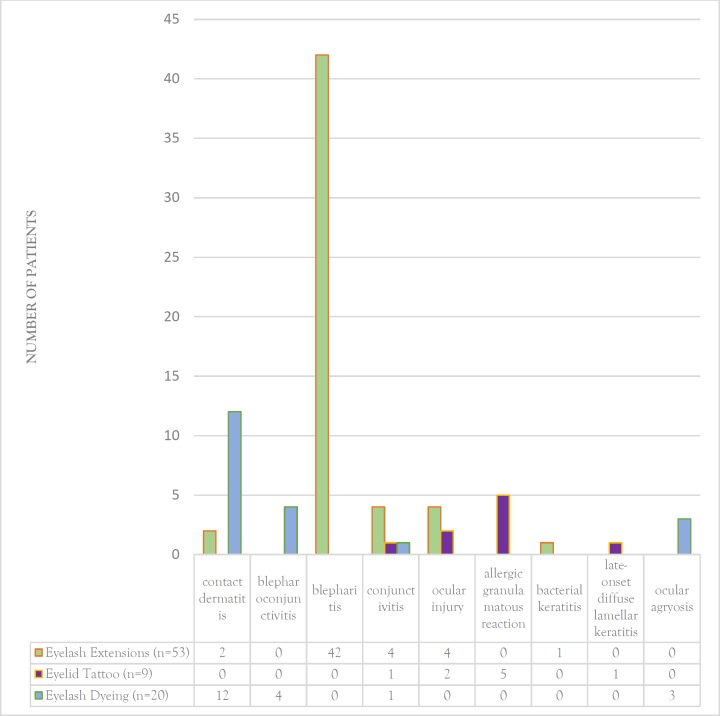
Number of Patients with the most Common Complications Following Eyelash Extensions, Eyelid Tattooing, and Eyelash Dyeing. Methodology for Findings Include Quantifying the Number of Associated Complications Reported in the Literature for Each Procedure.

Given the literature regarding enhancements to the eyelashes and eyelids, the potential high cost of modern society perception of beauty is evident (Fig. 6). The most commonly reported ocular disorders associated with each cosmetic procedure are summarized in Table 1. These alterations to the periorbital area have shown to be major contributors to ocular surface inflammatory disease, chronic dry eye syndrome and changes in visual acuity. Multiple studies in the literature have linked the toxicity of the substances used in each of the discussed cosmetic procedures with mild to severe ocular adverse effects. Amano et al [10] reported 42 cases of allergic blepharitis due to a formaldehyde-containing resin adhering eyelash extensions, in a Japanese study from March 2007 to 2010. The amount of formaldehyde detected in the cyanoacrylate was well above the safe cosmetic standard set by the Japanese Pharmaceutical Affairs Law, which was postulated to have caused the allergic blepharitis [10].

Another case report by Schwarze et al [27] presented a case of delayed-hypersensitivity granulomatous reaction following blepharopigmentation. Histological examination of the right eye revealed infiltration with granulomatous reaction in the upper dermis inferred to be caused by the pigment deposited, containing aluminum-silicate [27]. Later Vogel et al [20] reported a case of blepharoconjunctivitis and severe allergic contact dermatitis after an eyelash dyeing procedure. The severe reaction was suspected to be caused by one of the components in the eyelash dye. The cause of the blepharoconjunctivitis and allergic contact dermatitis was confirmed following a positive reaction patch test for P-phenylene diamine [20]. The glues, ink pigments and dyes applied during their associated ocular cosmetic procedures have proven to carry high risks of allergic reaction and damage to the ocular and periorbital surface given their toxic ingredients. Patch testing, when done properly may reduce the amount of adverse effects associated with these procedures, but has been reported to be insufficient in predicting tattoo reactions [27].

Physical application and mechanical implications of eyelash extensions and eyelid tattooing have been reported in the literature to accentuate adverse effects to the ocular surface. Lee et al. [5] investigated changes of meibomian gland and tear film instability in patients with eyelid tattoos. The study revealed meibomian gland loss was high in patients with eyelid tattoos because of the mechanical damage caused by the needle, toxicity of the tattoo substance often containing P-phenylene diamine and misapplication of the ink into a meibomian gland duct. Patients with eyelid tattoos also reported to have a lower tear secretion volume inferring tear film instability [5]. Moshirfar et al. [17] presented a case of inadvertent pigmentation of the limbus during blepharopigmentation causing pigment deposition into the conjunctiva, superficial cornea and sclera. Surgical removal of the pigment was performed to avoid chronic inflammation of the conjunctiva [17].

Koffuor et al. [9] studied mechanical limitations to eyelash extensions and reported that reduced blinking, lagophthalmos during sleep, misdirection of lashes falling into the eyes, and difficulty removing the lashes led to multiple ocular irritations and diseases [9]. Eyelash extension and eyelid tattooing procedures may result in mild to severe injury if misapplied or adhered incorrectly. Thus, such procedures should be performed by licensed professionals with sufficient knowledge of associated complications.

**Table:** 

Cosmetic Ocular Procedures	Commonly Associated Ocular Disorders
**Eyelash Extensions**	Allergic blepharitis, contact dermatitis, conjunctival erosion, subconjunctival erosion, bacterial keratitis, and keratoconjunctivitis
**Permanent Eyelid Tattooing**	Allergic granulomatous reactions, conjunctivitis, late-onset diffuse lamellar keratitis, inadvertent pigmentation of the limbus, eyelid penetration, tear film instability, and Meibomian gland loss
**Eyelash Dyeing**	Contact dermatitis, periorbital dermatitis, edema of the lids and erosions, and ulcerations of the cornea

The patients who underwent these procedures are more likely to have gone through other elective ocular surgeries like laser in-situ keratomileusis (LASIK), small incision lenticule extraction (SMILE), and refractive lens exchange (RLE). A past history of refractive surgery can potentially heighten the risk of developing ocular complications following these enhancements. Few case reports exist in the literature regarding the association between history of refractive surgery and cosmetic procedural complications. For example, Lu et al. [28] reported a case of bilateral diffuse lamellar keratitis triggered by permanent eyelid tattooing in a patient who had undergone LASIK 10 years before. The late-onset DLK was thought to be provoked by a hypersensitive reaction to the ink pigments producing granular infiltrates surrounding the edges of the corneal flaps [28]. 

Millions of these procedures are performed annually without any adverse effects. These procedures can be safe and effective when performed by a licensed practitioner. Since most of these techniques require use of toxic substances and disruption of healthy tissue, short-term damage may follow; however, limited literature is available on the long-term effects of these procedures. It is crucial for clinicians to understand the potential adverse effects of these cosmetic enhancements given the damage they can inflict to the ocular surface and how they can impact visual outcomes. Continued research is warranted due to the rise in popularity of these cosmetic enhancements and the potential consequences associated with each of them.

## CONCLUSIONS

The most prevalent cosmetic enhancements today including eyelash extensions, eyelid tattooing, and eyelash dyeing are rapidly gaining popularity worldwide. The rise in demand for such procedures warrants the need for proper education and training on the associated risks and complications. Review of the literature discussing these procedures shows an evident rise in complications linked to toxic substances involved, misapplication and a history of refractive surgery prior to cosmetic enhancement resulting in increased incidence of complications. Clinicians should be aware of the potential complications associated with these procedures, to better educate their patients on the role these procedures may play in their overall eye health, and to initiate guidelines that may ensure success in refractive correction. 
